# *Total Saikosaponins of Bupleurum yinchowense* reduces depressive, anxiety-like behavior and increases synaptic proteins expression in chronic corticosterine-treated mice

**DOI:** 10.1186/s12906-018-2186-9

**Published:** 2018-04-02

**Authors:** Xiuping Sun, Xianglei Li, Ruile Pan, Yanfeng Xu, Qiong Wang, Mingjing Song

**Affiliations:** 1 Comparative Medical Center, Beijing Key Laboratory for Animal Models of Emerging and Reemerging Infectious Diseases, Peking Union Medical College (PUMC) & Institute of Laboratory Animal Science, Chinese Academy of Medical Science(CAMS), PanjiayuanNanli No. 5, Beijing, 100021 China; 2 Institute of Medicinal Plant Development, Chinese Academy of Medical Science, Peking Union Medical College, No 151, North Road Malianwa, Haidian District, Beijing, 100193 China; 3 Prelinical Medicine Research Center/School of Pharmacy, Southwest Medical University, Zhongshan Road, 3-319, Jiangyang district, Luzhou, 646000 Sichuan Provience China

**Keywords:** Depression, Anxiety, AMPA receptor, Corticosterone, *Bupleurum yinchowense*, Synaptic protein

## Abstract

**Background:**

*Bupleurum yinchowense Shan et Y. Li* is widely used to treat depressive and anxiety disorders for hundreds of years in China. Total saikosaponins (TSS) is the major ingredient of *Bupleurum yinchowense*. A-amino-3-hydroxy-5-methyl-4-isoxazolepropionic acid (AMPA) receptor and subsequent mammalian target of rapamycin (mTOR) signaling is responsible for synaptic maturation and may contribution to the synaptic alteration underlying depression. The aim of the study was to investigate the antidepressant-like and anxiolytic effect of TSS in chronic corticosterone-treated mice. The effect of TSS on synaptic proteins expression and AMPA receptor-mTOR signaling pathway alteration was also evaluated.

**Methods:**

Dose-response effect of TSS (12.5, 25, 50 mg/kg) was investigated in forced swim test (FST) in ICR male mice. In the chronic corticosterine-treated model, TSS was given intragastrically once a day for 2 weeks and continued through the behavior testing period. Behavior tests and AMPA receptor related signaling pathway were investigated.

**Results:**

TSS (25 and 50 mg/kg) decreased the immobility time in the FST when compared with the control group. TSS (25 mg/kg) showed antidepressant-like and anxiolytic effects in the chronic corticosterone treatment model in mice. TSS increased hippocampal synaptic proteins (synapsin-1 and postsynaptic density protein 95) expression. Immunohistochemistry analysis showed that TSS significantly increased the synapsin-1 expression in CA3 of hippocampus. TSS also increased hippocampal phosphorylation expression of GluR1 Ser 845 (AMPA receptor subunit) and its downstream regulators extracellular signaling-regulated kinase (ERK), protein kinase B (Akt) and mTOR.

**Conclusion:**

TSS produces antidepressant-like and anxiolytic effects and increases synaptic proteins expression which may be mediated by induction of AMPA receptor and subsequent mTOR signaling pathway.

## Background

*Bupleurum yinchowense Shan et Y. Li*, a widely used Chinese medicine, was first documented in the*“Shennong’s Herbal”, which is the oldest Chinese materia medica monographs.* It was recorded that *Bupleurum yinchowense* has action of soothing liver and was *used* to treat depressive and mood related disorders [1]. Our preliminary experiments indicated that total saikosaponins (TSS) from *Bupleurum yinchowense* produced antidepressant-like effect in the forced swim test (FST), tail suspension test in normal mice. Meanwhile, TSS exhibited antidepressant-like effect in chronic mild stress model in mice [2]. However, the underlying mechanisms of these effects are not yet clear.

Recently, clinical and basic studies demonstrate that depression is associated with synaptic dysfunction in important regions such as prefrontal cortex and hippocampus [3]. Simultaneously, accumulating evidence has shown that A-amino-3-hydroxy-5-methyl-4-isoxazolepropionic acid (AMPA) receptor and subsequent mammalian target of rapamycin (mTOR) signaling way is responsible for the induction and maintenance of synaptic plasticity and may contribution to the synaptic alteration underlying depression [4, 5]. Chronic AMPA potentiators and AMPA treatment produce antidepressant-like effect and stimulate mTOR protein transduction signaling way [6]. The mTOR singaling is a downstream intracellular signal pathway which controls the synthesis of synaptic proteins and contributes to synaptogenesis [7]. Ketamine fast induction of mTOR singaling and synaptogenesis may be dependent on AMPAR activation [8]. Sarcosine, an endogenous amino acid, exhibits antidepressant-like effects by activating AMPAR–mTOR signaling pathway [9]. These data suggest that AMPA receptors and subsequent mTOR signaling may be a potential therapeutic target for emerging antidepressant-like agent actions.

Chronic stress-mediated elevated levels of circulating glucocorticoids (corticosterone, CORT, in rodents) are widely believed to be involved in the pathophysiology of depression [10]. Chronic and direct administration of corticosterone to male rodents has been used as a model to induce anxiety/depression-like behavior [11, 12]. In such models, hippocampal dendritic spines and spine synapses were decreased which is accompanied by a loss of key synaptic proteins such as postsynaptic density protein 95 (PSD-95), and the presynaptic protein synapsin-1 [12]. Interestingly, some findings indicated CORT exerted its impact on synaptic plasticity by modulating AMPA receptor [13].

Our recent research showed that TSS induced protective effect in corticosterone-induced damage in PC12 cells [14]. On the basis of above findings, we hypothesize that chronic CORT treatment-induced depressive and anxiety like behaviour of mice is caused by dysregulation of AMPA receptor and subsequent mTOR signaling pathway and treatment with TSS might ameliorate these behavioural and molecular changes.

## Methods

### Preparation of Total Saikosaponins (TSS)

TSS was extracted as previous prescribed [14]. Five main monomeric compounds in TSS were determined using HPLC and the content of Saikosaponins a, c, d, e and f was 10.12%, 2.84%, 14.13%, 1.52% and 2.14%, respectively.

### Animals

ICR and C57BL/6J male mice (23–25 g) were obtained from Beijing HFK Bioscience CO., LTD. The animals were group-housed (5 per cage) under the standard conditions (25 °C with a 12 h/12 h light/dark cycle) and received standard food and tap water ad libitum. The experiments were approved by the ethical committee for the use of experimental animals of the Institute of Laboratory Animal Sciences (No: ILAS-PG-2015-011).

### Drugs

Citalopram was purchased from the National Institutes for Food and Drug Control (Beijing, China). Citalopram were dissolved in distilled water. Corticosterone (CORT, from Sigma, 27,840) was dissolved in vehicle (0.45% hydroxypropyl-β-cyclodextrin, β-CD from Binzhou Zhiyuan Bio-Technology, China).

### Experiment 1 dose-response effect of TSS in the FST

Forty-one ICR male mice were divided randomly into control (distilled water, *n* = 10), TSS (12.5 mg/kg, *n* = 10), TSS (25 mg/kg, *n* = 10) and TSS (50 mg/kg, *n* = 11) groups. TSS or distilled water was given orally 1 h before testing. FST is the most popular behavioral test to evaluate depression-like behavior in rats and mice, with a high predicative validity for screening antidepressant drugs [15]. According to the method described by Porsolt, the apparatus consisted of a plastic cylinder (height 20 cm, diameter 12 cm) filled with water (25 °C) to a depth of 15 cm. Mice were individually placed into the cylinder for 6 min. The immobility time was recorded during the last 4-min interval of the test by trained observers who were blind to the treatment of mice. Each mouse was judged to be immobile when it ceased struggling and made only small movements to keep its head above the water [15].

### Experiment 2 anxiolytic, antidepressant-like effects and biochemical alteration of TSS in chronic corticosterone-treated mice

**Fig. 1:**
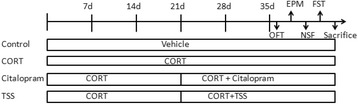
The schematic representation of experiment 2 design. Male C57BL/6 J mice were divided 4 groups: Control, CORT, Citalopram (10 mg/kg) and TSS (25 mg/kg). Vehicle and corticosterone were delivered for 5 weeks and continued through the behavior testing period. After 3 weeks of drug-free exposure to CORT administration, TSS and citalopram were given intragastrically once a day for 2 weeks and continued through the behavior testing period. OFT, EPM, NSFT and FST were performed. After behavior tests, the mice were sacrificed for western blot essay or immunohistochemistry analysis

To assess the depressive, anxiety behavior and molecular alteration of TSS administration in chronic corticosterone-treated mice, male C57BL/6 J mice were randomly divided into 4 groups (*n* = 8–9 per group): Control (*n* = 9), CORT (*n* = 8), citalopram (10 mg/kg, *n* = 8)) and TSS (25 mg/kg, *n* = 9). Corticosterone (35 μg/ml equivalent to 5 mg/kg/day) was delivered for 35 days in drinking water in opaque bottles and continued through the behavior testing period. Control animals received vehicle (β-CD) in drinking water during the entire experiment. After 3 weeks of drug-free exposure to CORT administration, TSS was given intragastrically once a day for 14 days and continued through the behavior testing period. Citalopram was used as positive control. Open field test (OFT), elevated plus maze (EPM) test, novelty suppressed feeding test (NSFT) and forced swim test (FST) were performed to assess anxious-depressive like behavior (Fig. 1).

### Behavior tests

#### Open field test (OFT)

Anxiety behavior and motor activity was quantified in an open field paradigm (50 × 50 × 50 cm) using a computerized video-tracking system (EthoVision XT,Noldus). Animals naturally tend to avoid the central parts of open field. Anxiety state was assessed by the time spent in the center area of open field. In addition, OFT is routinely used to assess spontaneous locomotor activity in rodents [16].The open field was divided into center, transition and border arena. The behavior experimenters were blind to the treatment of mice. Each mouse was gently placed in the center of the box. The total distance moved, time in the center area and velocity were recorded for 5 min and analyzed by automated system software to evaluate the locomotor activity and anxiety-related behavior.

#### Elevated plus maze (EPM) test

EPM test has widely been used for screening anxiolytic drugs. The animals spontaneously prefer the closed arms to open ones. Anxiety state was assessed by the percentage of open arm entries and percentage of time spent on the open arms [17].The maze was made of two open arms (30 cm × 5 cm) and two closed arms (30 cm × 5 cm × 15 cm) and it was elevated 50 cm above the floor. The intersection of the arms was a central platform (5 cm × 5 cm). The behavior experimenters were blind to the treatment of mice. The mouse was placed in the central platform with its head facing an open arm. After 5 min of free exploration, the mouse may be moved out of the maze and back into its home cage. The number of closed arm entries, number of open arm entries, and time spent on the open arms were recorded and analyzed by automated computer software (EthoVision XT,Noldus). The percentage of open arm entries (%OE = 100 × open arm entries / total entries) and percentage of time spent on the open arms (%OT = 100 × time spent on open arms / (time spent on open arms + time spent on closed arms) were calculated.

#### Novelty suppressed feeding test (NSFT)

NSFT has been used extensively to study anxiety and depression related behaviors since it is sensitive to anxiolytic and chronic antidepressant treatments [18].The apparatus consisted of a plastic box (50 cm × 50 cm). After 24 h of food deprivation, a single pellet of food was placed in the center of the box. The animal was placed in a corner of the box and the latency to bite was recorded for 5 min by trained observers who were blind to the treatment of mice. The home-cage food consumption during the subsequent 5 min was measured.

### Western blotting analysis

At 24 h following the behavior testing, animals were killed by decapitation, brains were removed and hippocampus was dissected out for western blot experiments. The dissected tissue was made in the RIPA lysis buffer with protease and phosphatase inhibitor cocktail (Cell Signaling Technology). The protein concentration in each sample was determined using a Pierce BCA protein assay kit. The proteins were separated on SDS-PAGE gel and transferred onto a nitrocellulose membrane. The membranes were blocked with the blocking reagent for 2 h and incubated at 4 °C overnight with the primary rabbit antibodies against phospho-mTOR (Ser-2448), phosohpo-ERK1/2 (Thr202/Tyr204), phospho-Akt (Ser-473), synapsin-1, PSD-95 (Cell Signaling Technology), phospho-GluR1 (Ser-845) (Abcam). Membranes were subsequent incubated for 1 h at room temperature in goat anti-rabbit HRP-conjugated secondary antibody (1:5000 in TBS, ZSGB-BIO, China). Immunoreactive bands were visualized using enhanced chemiluminescent kits (Santa Cruz).

### Immunohistochemistry analysis

For immunohistochemistry, mice were anesthetized with sodium pentobarbital (50 mg/kg body weight, i.p.) and perfused with a 4% paraformaldehyde solution. Tissues were routinely processed in paraffin blocks. Immunohistochemical staining was performed according to Polink-2 plus System kit (ZSGB-BIO, China). Sections were blocked in10% normal goat serum and were subsequently incubated in synapsin-1primary antibody solution (1:1000) at 4 °C overnight. Sections were washed in PBS with 0.3% Triton X-100 and incubated in goat anti-rabbit secondary antibody (1:1000) for 1 h at room temperature. Images were taken with a BX40 microscope with an attached CCD camera system (Olympus, Tokyo, Japan). Image quantification was analyzed using Image-pro Plus software.

### Statistical analysis

SPSS 20 was used for statistical analysis. Differences among means were analyzed using one-way analysis of variance (ANOVA) followed by the Student-Newman-Keuls test. All results are presented as mean ± standard error of the mean (SEM). *P* < 0.05 was considered statistically significant for all data.

## Results

### TSS produced antidepressant-like effect in the FST in mice

**Fig. 2:**
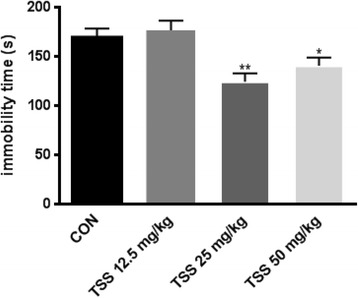
Effects of TSS (12.5、25、50 mg/kg) on the forced swim test. Data were analyzed by one-way ANOVA followed by Student-Newman-Keuls test. Each bar represents the mean ± SEM (*n* = 10–11). **P < 0.01,^*^P < 0.05, as compared with the control group

As shown in Fig. 2, TSS, at dose of 25 and 50 mg/kg, decreased the immobility time in the FST when compared with the control group (F(3,37) = 7.512, *P* < 0.01; *P* < 0.05). Dose of 12.5 mg/kg did not produce significant effect.

### TSS produced antidepressant and anxiolytic effects in the chronic cortiocosterone-treated mice

#### Open field test

**Fig. 3:**
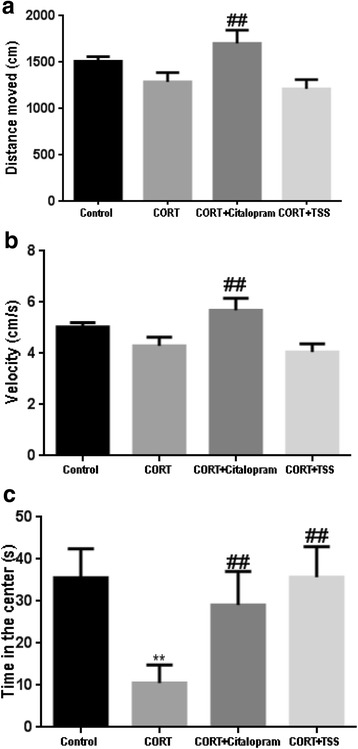
Effects of TSS (25 mg/kg) and citalopram (10 mg/kg) on the OFT in the chronic CORT treatment mice. **a** Distance moved. **b** Velocity. **c** Time in the center. Data were analyzed by one-way ANOVA followed by Student-Newman-Keuls test. Each bar represents the mean ± SEM (*n* = 8–9). **P < 0.01, as compared with the control group and ^##^*P* < 0.01, as compared with the CORT group

As shown in Fig. 3a, b, c, no significant difference in the distance moved and velocity was found between CORT group and control group (F(3,30) = 4.314, both *P* > 0.05). Citalopram significantly increased the velocity and distance moved compared with CORT group (both *P* < 0.01). TSS treatment did not produce any change in the distance moved and velocity (both *P* > 0.05). Both citalopram and TSS significantly increased the center time in the OFT which is decreased by CORT treatment (F(3,30) = 3.534,both *P* < 0.01).

#### Elevated plus maze test

**Fig. 4:**
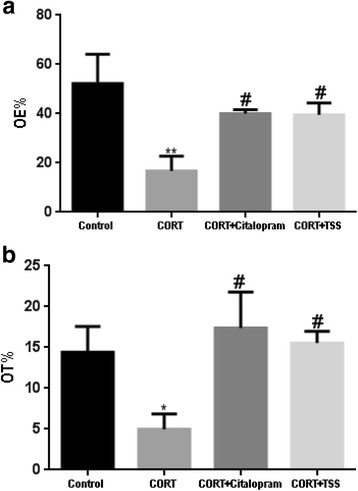
Effects of TSS (25 mg/kg) and citalopram (10 mg/kg) on the EPM test in the chronic CORT treatment mice. **a** the percentage of open arm entries (OE%). **b** the percentage of time on the open arms (OT%). Data were analyzed by one-way ANOVA followed by Student-Newman-Keuls test. Each bar represents the mean ± SEM (*n* = 8–9). **P < 0.01, *P < 0.05 as compared with the control group and ^#^*P* < 0.05, as compared with the CORT group

As shown in Fig. 4a and b, chronic corticosterone treatment induced significant reduction in the percentage of open arm entries and percentage of time on the open arms (F(3,30) = 3.527, *P* < 0.01; F(3,30) = 2.913,*P* < 0.05) compared with control group. Both citalopram and TSS treatment reversed chronic CORT treatment induced changes in the EPM test (both *P* < 0.05).

#### Novelty suppressed feeding test

**Fig. 5:**
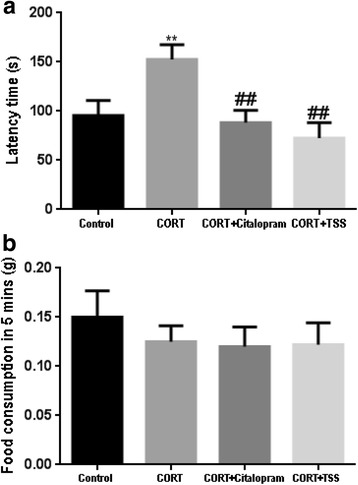
Effects of TSS (25 mg/kg) and citalopram (10 mg/kg) on the NSFT in the chronic CORT treatment mice. **a** Latency time (s). **b** Food consumption in 5 mins. Data were analyzed by one-way ANOVA followed by Student-Newman-Keuls test. Each bar represents the mean ± SEM (*n* = 8–9). **P < 0.01, as compared with the control group and ^##^*P* < 0.01, as compared with the CORT group

As shown in Fig. 5a and b, chronic corticosterone treatment significantly increased the latency time to feed in the NSF test with respect to the control group (F(3,30) = 6.927, *P* < 0.01). Both citalopram and TSS significantly reduced the latency time (both *P* < 0.01). No differences in home-cage feed consumption were detected (both *P* > 0.05).

#### Forced swim test

**Fig. 6:**
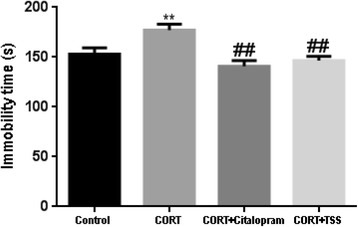
Effects of TSS (25 mg/kg) and citalopram (10 mg/kg) in the FST in the chronic CORT treatment mice. Data were analyzed by one-way ANOVA followed by Student-Newman-Keuls test. Each bar represents the mean ± SEM (*n* = 8–9). **P < 0.01, as compared with the control group and ^##^*P* < 0.01, as compared with the CORT group

As shown in Fig. 6, chronic corticosterone treatment significantly decreased the immobility time in the FST compared with control group (F(3,30) = 6.547, *P* < 0.01). Both citalopram and TSS significantly increased the immobility time (both *P* < 0.01).

### TSS improved AMPA receptor-mTOR signaling pathway and synaptic proteins expression in the hippocampus of chronic corticosterone-treated mice

#### TSS improved AMPA receptor-mTOR signaling pathway

**Fig. 7:**
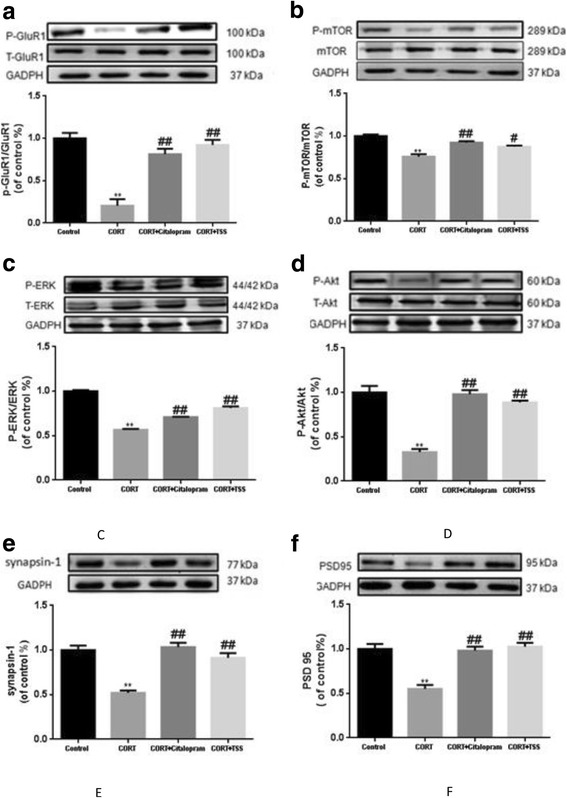
Representative western blot analysis of P-GluR1(**a**), P-mTOR (**b**), P-ERK (**c**), P-Akt (**d**), synapsin-1(**e**) and PSD-95 (**f**) in the hippocampus after TSS (25 mg/kg) and citalopram (10 mg/kg) treatment in chronic corticosterone-treated mice. Data were analyzed by one-way ANOVA followed by Student-Newman-Keuls test. Each bar represents the mean ± SEM. **P < 0.01, as compared with the control group and ^##^*P* < 0.01, ^#^*P* < 0.05, as compared with the CORT group

As shown in Fig. 7a, chronic corticosterone treatment significantly decreased the phosphorylation of hippocampal GluR1 Ser845 in the hippocampus in chronic corticosterone-treated mice (F(3,8) = 48.83, *P* < 0.01), which was reversed by the citalopram and TSS treatment (both *P* < 0.01). Total GluR1 level remained unchanged. Similarly, the results in Fig. 7b, c, d showed chronic corticosterone treatment significantly decreased the phosphorylation of mTOR, ERK and Akt in the hippocampus of chronic corticosterone-treated mice (F(3,8) = 46.636; F(3,8) = 22.880; F(3,8) = 29.458, both *P* < 0.01). Citalopram and TSS treatment significantly increased the p-Akt (both *P* < 0.01), p-ERK (*P* < 0.05, *P* < 0.01) and p-mTOR level (*P* < 0.01, *P* < 0.05).

#### TSS improved synaptic proteins expression

As shown in Fig. 7e, f, chronic corticosterone treatment significantly decreased synapsin-1 and PSD-95 level in the hippocampus in chronic corticosterone-treated mice (F(3,8) = 37.245; F(3,8) = 30.224; both *P* < 0.01). Citalopram and TSS treatment significantly increased synapsin-1 and PSD-95 level (both *P* < 0.01).

### Immunohistochemical analysis of synapsin-1 in the hippocampus of chronic corticosterone-treated mice

**Fig. 8:**
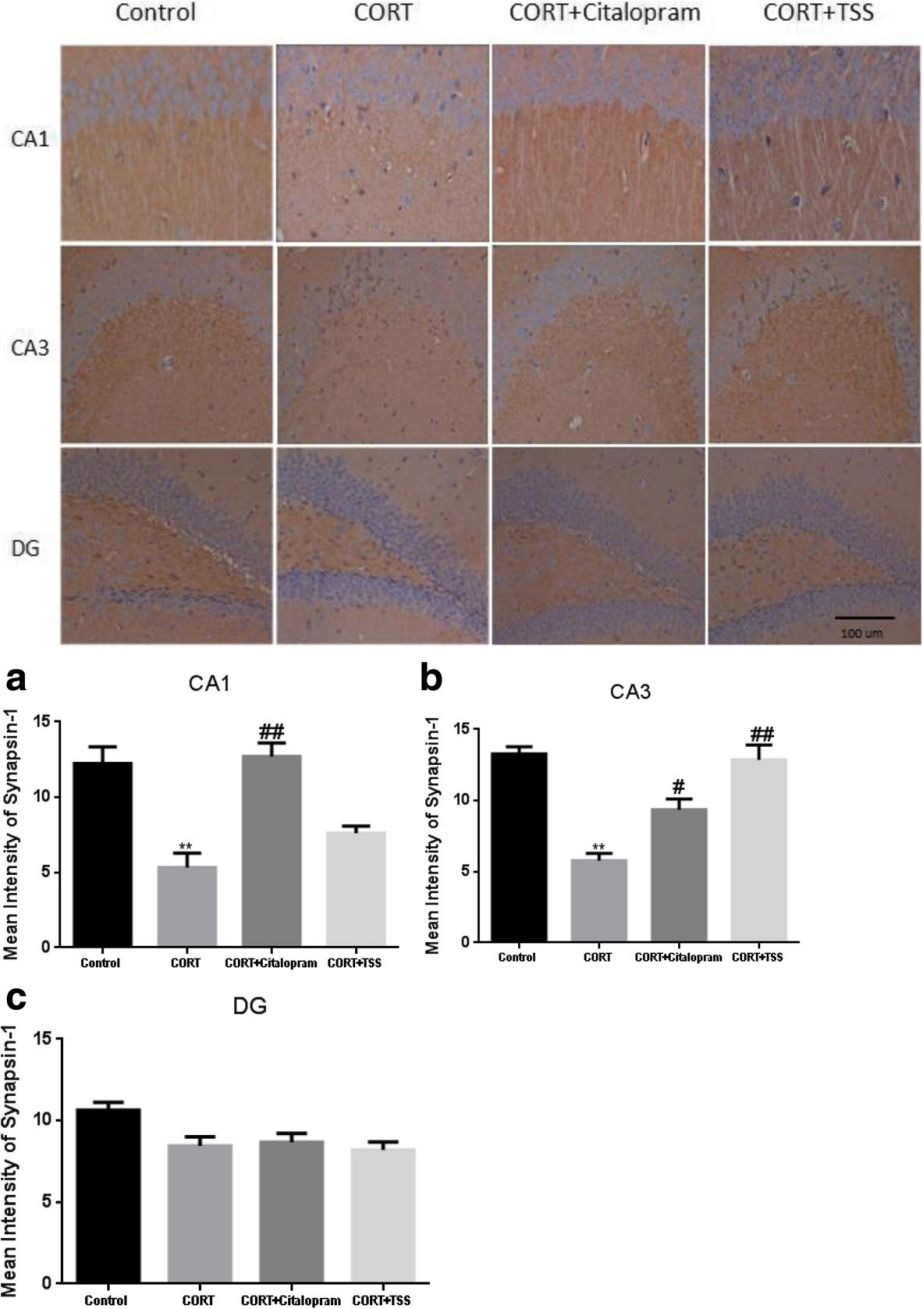
Immunohistochemical analysis of synapsin-1 in the CA1 (**a**), CA3 (**b**) and  DG (**c**) of hippocampus after TSS (25 mg/kg) and citalopram (10 mg/kg) treatment in chronic corticosterone-treated mice. Data were analyzed by one-way ANOVA followed by Student-Newman-Keuls test. Each bar represents the mean ± SEM. **P < 0.01, as compared with the control group and ^##^*P* < 0.01, ^#^*P* < 0.05, as compared with the CORT group

To further assess the effect of TSS on the expression of synapsin-1 in the hippocampus of chronic corticosterone-treated mice, immunohistochemical analysis was performed. Consistent with western blotting analysis results, as shown in Fig. 8, in the Cornu Amonis (CA1) (**a**) and CA3 (**b**), chronic CORT treatment mice displayed significantly less levels of synapsin-1, compared with control group (F(3,8) = 16.269; F(3,8) = 22.305; both *P* < 0.01). In the dentate gyrus (DG) region (**c**), chronic CORT treatment did not cause a significant decrease in synapsin-1 expression (F(3,8) = 4.563, *P* > 0.05). TSS significantly increased the synapsin-1 expression in the CA3 region (*P* < 0.01), but not in the CA1 and DG of hippocampus (both *P* > 0.05). Citalopram significantly increased the synapsin-1 expression in the CA1 and CA3 region (*P* < 0.01, *P* < 0.05). No significant change was observed after citalopram treatment in the DG region (*P* > 0.05).

## Discussion

The present study indicates TSS exerts antidepressant-like and anxiolytic effects that are common to citalopram in chronic corticosterone-treated mice. Moreover, TSS increases synaptic proteins (synapsin-1 and PSD-95) which may be mediated by activation of AMPA receptor and mTOR signaling pathway. Dose-response assessment shows TSS produces antidepressant-like effect in the FST both in 25 and 50 mg/kg CORT-treated mice. Notably, the dose of 25 mg/kg presents maximum anti-immobility effect in the FST. Our previous work has shown that TSS at dose of 25 mg/kg was more efficacious than that of 50 mg/kg at different time point post the last TSS administration [19]. Previous study also indicated saikosaponin at dose of 25 mg/kg produced antidepressant-like effects in the chronic mild stress model [20]. Herein, the dose of 25 mg/kg was selected to further research.

To evaluate behavioral changes, in the present study, OFT, EPM, NSFT and FST were used to assess anxious-depressive like behavior of the chronic corticosterone treatment model in mice. Our study showed that 5 week administration of CORT at dose of 35 μg/ml per day induced depressive like behavior as reflected by the increase in the immobility time in the FST. This model also exerted anxiety behavior as assessed by the decrease in the center area in the OFT and by the decreased percentage of open arm entries and percentage of time spent on the open arms in EPM test, as well as by the increased latency to feed in the NSFT. Thus, in this study, an anxiety-depressive model was successfully modeled by chronic treatment of CORT. These behavior results obtained from our study are consistent with previous findings [11, 21, 22].

Depressive and anxiety disorder commonly occur together in clinic [23]. Clinic research has shown that more than 70% patients with depressive disorder present symptoms of anxiety disorder [24]. Co-morbid depression and anxiety increase suicide risk and contribute to treatment resistance [25]. The selective serotonin reuptake inhibitors (SSRIs) are usually used to treat co-morbid depression with anxiety, although there are drawbacks including sexual and weight gain. In the present study, it is the first time to detect the anxiolytic and antidepressant-like effect of TSS in chronic CORT treatment model by behavior tests such as OFT, EPM, NSFT and FST. Citalopram, a classic prototypical selective serotonin reuptake inhibitor was the positive control. Our results showed that both TSS and citalopram exerted anxiolytic effect, as measured by a significant increase of time spent in the center of the open field and the increased percentage of open arm entries and percentage of time spent on the open arms in the EPM test, as well as by the decreased latency to feed in the NSFT. Furthermore, TSS was effective in reducing the immobility time in the FST, suggesting an antidepressant-like effect. Since locomotor activity may lead to false positive or negative effects in the FST, OFT was used to assess the locomotor activity of animals. Our research has shown that TSS did not produce significant changes in locomotion activity in the OFT. Therefore, the effect of TSS in the FST was not based on the unspecific action such as stimulation of general motor. Whereas, the mice treated with citalopram increased the distance moved and velocity, which is in line with previous study [26]. Taken together, TSS and citalopram exerted anxiolytic and antidepressant-like effects in chronic CORT treatment model. Our preliminary experiment indicated treatment with TSS produced a significant increase of 5-HT level in the brain as compared to chronic mild stress model in mice. Increasing the 5-HT level in the brain may be the common path of antidepressant-like effect of citalopram and TSS. Glucocorticoid receptor (GR) mediates the expression of the serotonin transporter (SERT) and increases the 5-HT level in the brain. We have previous shown that Saikosaponins D, the main active ingredient of TSS, up-regulated the GR protein expression in corticosterone-induced PC12 cells. This result suggests that TSS induced increasing 5-HT level in the brain may be mediated by activating GR [14]. In the subsequent study, we should analyze the alteration of GR in the brain in the chronic corticosterone treatment model and investigate the effect of TSS on the GR expression.

It has been postulated that synaptic plasticity and synaptogenesis play an important role in the pathogenesis of depression and mechanisms of action of current antidepressant drugs [3]. Synapsin-1 is a presynaptic marker and is widely used for measurement of synaptic density [27]. PSD-95 is a post synaptic marker and is involved in glutamatergic synaptogenesis [28]. To detect whether costicosterone decreased the synaptic proteins level, synapsin-1 and PSD-95 levels were assessed in the hippocampus of chronic corticosterone-treated mice. Our present western blotting analysis shows that chronic treatment costicosterone decreases synapsin-1 and PSD-95 level in mice. TSS and citalopram treatment shows an increase in synapsin-1 and PSD-95 levels in the hippocampus of mice exposed to 5 week CORT treatment. To reveal localized changes of synaptic density in the hippocampus, immunohistochemical analysis was used to determine synapsin-1 expression in sub-regions of hippocampus in CORT-treated mice. The present study shows that 5 week CORT treatment induces reduction in synapsin-1level in the CA1 and CA3 of hippocampus. This finding supported previous work which exhibited prolonged corticosterone exposure decreased dendritic spine densities in the hippocampus CA1 and CA3 [29]. In the previous studies, saikosaponin treatment induced molecular changes in the CA3 of hippocampus in the depression models [30, 31]. Consistent with these findings, we found TSS induced a pronounced increase of synapsin-1 expression in the CA3 of hippocampus. Citalopram increased synapsin-1 level both in the CA1 and CA3 region are in agreement with previous study showing that citalopram reversed the decreased synaptic plasticity in the hippocampus in the social isolation model [32]. These results suggest that TSS and citalopram might increase synaptic proteins expression and promote synaptogenesis in the hippcampus. Based on the above mentioned data, the mechanisms by which TSS and citalopram exert their antidepressant-like effects maybe involve both monoamine neurotransmission and synaptogenesis. Further studies should be performed to confirm this hypothesis.

Accumulating evidence shows that AMPA glutamatergic activation triggers the synaptogenic process in important regions such as the prefrontal cortex and hippocammpus [7]. In addition, several research results have shown that phosphorylation of mTOR and kinases ERK and Akt, is important mechanism underlying the activation of AMPA receptors in synaptogenesis [33–35]. Therefore, AMPA receptor-mTOR signaling pathway may be involved in the synaptic alteration underlying depression. To determine whether the antidepressant and anxiolytic like effects of TSS was accompanied by the activation of AMPA receptor-mTOR signaling pathway, the phosphorylation of GluR1 Ser845 (AMPA receptor subunit), mTOR and mTOR upstream regulator proteins (ERK and Akt) were assessed in chronic corticosterone-treated mice. Our research showed that TSS reversed the decreased level of P- GluR1 (ser845) induced by 5 week CORT administration. Simultaneously, 5 week CORT administration decreased the expression level of p-mTOR, p-ERK and p-Akt in the hippocampus, which was reversed by TSS and citalopram treatment. This finding of citalopram is in agreement with previous study showing that activation of the mTOR signaling was implicated in the antidepressant-like effect of subchronic citalopram treatment in the chronic mild stress model [36]. Our another study showed that pretreatment with 2,3-dioxo-6-nitro-1,2,3,4-tetrahydrobenzo (f) quinoxaline-7-sulfonamide (NBQX), an AMPA receptor antagonist, blocked the TSS induced antidepressant-like effects of decreased immobility time in the FST (data not shown). These results indicated that AMPA receptor-mTOR signaling pathway may be involved in the antidepressant-like effect of TSS. Further research is needed to examine whether pretreatment with NBQX would affect the activity of the mTOR signaling pathway (p-mTOR, p-ERK and p-Akt) after TSS treatment.

## Conclusion

The present study indicates that repeated administration of TSS exerts antidepressant-like and anxiolytic effects in chronic CORT administration mice. Our data provide evidence that TSS improves the depressive and anxiety like behavior via induction of AMPA receptor and subsequent mTOR signaling pathway which are involved in synaptogenesis. Together with our previous research, TSS could be a potential efficacious candidate as a new therapy for depression.

## Abbreviations

AMPA: A-amino-3-hydroxy-5-methyl-4-isoxazolepropionic acid
CORT: Corticosterone
EPM test: Elevated plus maze test
FST: Forced swim test
NSFT: Novelty suppressed feeding test
OFT: Open field test
TSS: Total saikosaponins
